# Clinician- and Patient-Centred Outcomes of Digital Impressions in Infants with Cleft Lip and Palate: A Systematic Review

**DOI:** 10.3390/children11030343

**Published:** 2024-03-13

**Authors:** Jyotsna Unnikrishnan, Yasaman Etemad Shahidi, Mahmoud Bakr, Robert Love, Ghassan Idris

**Affiliations:** 1 School of Medicine and Dentistry, Griffith University, Gold Coast, QLD 4222, Australia; jyotsna.unnikrishnan@griffithuni.edu.au (J.U.); m.bakr@griffith.edu.au (M.B.); r.love@griffith.edu.au (R.L.); 2 Children’s Oral Health Service and Child Specialist Services, Metro North Hospital, Queensland Children’s Hospital, South Brisbane, QLD 4101, Australia

**Keywords:** cleft lip, cleft palate, infant, systematic review, digital dental impression technique, parents, clinicians, perception

## Abstract

This systematic review examines the effectiveness of digital impressions in infants with cleft lip and palate (CLP), focusing on accuracy, operator preferences, and parents’ perceptions. The PICO-formulated focused questions assessed the accuracy and operator preference of digital impressions compared to conventional impressions in infants with cleft lip and palate, while also exploring parents’ perceptions as patient-centred outcomes. Electronic and manual searches were conducted in five databases including PubMed, Scopus, Web of Science, Embase, and Cochrane Library; to acquire grey literature, Google Scholar was also consulted. Both experimental and observational studies that used digital impressions in the clinical care of infants with CLP were included. The Joanna Briggs Institute Critical Appraisal Checklist was used to assess the quality of the included studies. Out of 503 records, 12 studies met the inclusion criteria. The accuracy assessment included surface discrepancy and intra-arch measurements. Surface discrepancy studies showed variations in the premaxillary segments, while intra-arch measurements revealed no significant differences. Operators preferred digital impressions, citing reduced stress and streamlined workflows. Parents expressed a clear preference for digital over conventional impressions. The conclusions drawn were substantiated by weak evidence due to the limited number and the high risk of bias of the included studies. Challenges remain here, warranting continued research to enhance accuracy and assess parents’ preferences, ensuring optimal outcomes for infants with CLP.

## 1. Introduction

Cleft lip and/or palate (CLP) is the most common congenital malformation in the head and neck region, affecting approximately 7.94 per 10,000 live births worldwide [1]. The comprehensive care of children born with CLP demands a long-term multidisciplinary approach. A pivotal aspect of this care involves using study models for diagnostic purposes, the construction of oral appliances, and longitudinal treatment assessment. The World Health Organization (WHO) recommends that study models be recorded after birth, before lip surgical repair in infancy, and later in other stages at ages 5, 10, and 18 to 20 years for CLP patients [2].

Orthodontists play a vital part in the treatment of CLP patients. Early orthodontic intervention, referred to as early maxillary orthopaedics, which involves obtaining an oral impression, is a common treatment method for newborns with CLP [3]. The initial impression and the resulting naso-alveolar moulding (NAM) appliance set the foundation for comprehensive care and play a crucial role in the surgical precision required in CLP repair [3]. A key issue with conventional **oral** impression using alginate or rubber-based impression materials is the risk of airway obstruction in children in the early stage after birth [4]. This occurs due to airway obstruction while the impression material is inside the mouth, or it may be due to fragmentation of the impression material during removal and subsequent aspiration, leading to asphyxia and cyanotic episodes [4]. Taking the impressions for a newborn with CLP has been recommended to be conducted by trained orthodontists and with access to clinicians who can provide emergency assistance whenever it is needed in case of a respiratory emergency [4,5]. Additional challenges that have been documented encompass reduced levels of oxygen saturation during procedures [6], difficulties in obtaining precise impressions due to premaxilla mobility, and the poor registration of buccal extensions caused by a lack of orbicularis sphincter function [7]. Moreover, lack of cooperation of patients and limited mouth opening often result in a challenging impression-acquiring procedure, particularly in neonates and infants with cleft lip and palate [8].

Digital impressions using intraoral scanners at various stages of CLP care have recently been introduced [9,10,11]. For example, they help to create a digital workflow for fabricating NAM appliances that prepare the newborn for cleft repair surgery [12]. Digital impressions using intraoral scanners are used as diagnostic aids, for printing 3D models to assist in treatment planning [13], and for measuring the outcome of various treatment methods in patients with CLP [14].

Cleft lip and palate (CLP) can impact parents on the social and psychological levels, leading to parenting stress and psychological distress [15]. This can also affect the family’s quality of life (QoL), particularly during the initial year of a child’s life, especially near the primary surgery [16,17]. Hence, in this context, analysing the parent- reported outcome in terms of digital impressions in infants is critical, as it could be a contributing factor affecting the QoL. Similarly, clinicians require digital impressions that provide accurate and precise representations of the infant’s oral structures. It is imperative that the digital impressions are reliable and easy for clinicians to use. The efficiency of the digital impression process is crucial for clinicians who need to work quickly and effectively, especially with infants who may have limited tolerance for procedures. By optimising the workflow, the experience of the clinician and the patient can be significantly improved [12,14].

Although numerous reviews have investigated the comparison between digital and conventional models in various dental specialities [18,19,20], there remains a conspicuous research gap in our understanding of the efficacy of digital impressions in infants with CLP. Considering the potential airway obstruction risks of conventional impressions and the ongoing financial and human resources required to ensure an airway management team is onsite, an evidence-based recommendation in this space is critical. This systematic review aims to address this research gap by determining the efficacy of digital impressions in infants with CLP, focusing on accuracy, parent acceptance, and operators’ preference.

## 2. Materials and Methods

*Protocol and registration*

This systematic review followed the Cochrane Handbook for systematic reviews and interventions and adhered to the Preferred Reporting Items for Systematic Reviews and Meta-Analyses (PRISMA) guidelines. Before protocol registration with the National Institute of Health Research’s PROSPERO Protocol Registry [21], a comprehensive search was conducted, which failed to identify similar reviews. The protocol for this study was registered on the International Prospective Register of Systematic Reviews (https://www.crd.york.ac.uk/prospero, 18 December 2021) (PROSPERO Reg No: CRD42022298846).

*Focused Questions:*

The focused questions derived from the population, intervention, control, and outcome (PICO) framework were as follows:

*Clinician-Centred Questions:*

1. “For infants with cleft lip and palate, do digital impressions have similar accuracy to conventional impressions?”
2. “Do operators prefer digital impressions for infants with cleft lip and palate over conventional impressions based on their experience?”

*Parent-Centred Questions:*

1. “Do parents prefer digital impressions for infants with cleft lip and palate over conventional impressions?”

*PICO criteria:*

Participants: Non-syndromic male and female infants with unilateral or bilateral cleft lip and palate, irrespective of cleft severity.

Interventions: Digital impressions using intraoral scanners and direct digital models generated with intraoral scanners in infants with CLP.

Comparison: Conventional impressions using alginate, putty impression materials, or conventional plaster models.

Outcome:

*Clinician-Centred Outcome Measure(s):* Accuracy of digital impressions in infants with CLP compared to conventional impressions and operator preferences.
*Parents-Centred Outcome Measure(s):* Parents’ perceptions and preference for digital impressions in infants with CLP.

*Inclusion criteria:*

1. Studies that compared digital impressions or digital models derived from them with models from conventional impressions in infants with CLP were included for accuracy evaluation.
2. Clinical studies using digital or conventional impressions on infants with CLP were included for evaluating patient or clinician preferences.

*Study design:* All types of studies, such as randomised controlled trials, cohort studies, cross-sectional investigations (with and without controls), and case series and case reports involving the use of digital impressions in infants with CLP, were included in the initial search. There was no language restriction in the searches, and all relevant studies were included up until our search date (Last update: November 2023).

*Exclusion criteria:*

Studies focusing on craniofacial deformities other than CLP and those based on cone beam computed tomography were excluded from our analysis.

*Information sources, search strategy, and study selection:*
*Information sources:*

An electronic search was conducted by two independent reviewers (JU and YE) to identify relevant studies published before 25 November 2023 in five databases, PubMed, Scopus, Web of Science, Embase, and Cochrane Library, using the search criteria mentioned in Supplementary Table S1. The search criteria were developed to identify all studies related to digital impressions in infants. The bibliography of the included manuscripts was cross checked for relevant works and included when appropriate. In addition, a manual search was carried out in the *American Journal of Orthodontics and Dentofacial Orthopaedics*, the *European Journal of Orthodontics*, the *Angle Orthodontist*, and *The Cleft Palate-Craniofacial Journal* to locate any articles that might have been missed during the electronic database search.

*Search strategy and study selection:*

Details of the electronic search strategy in each database are provided in Supplementary Table S1. All relevant studies were uploaded to Covidence, and deduplication was carried out. The titles and abstracts were screened independently by two reviewers (JU and YE) using the eligibility criteria. Then, the full texts of the articles were independently identified and evaluated by the reviewers mentioned above to assess the degree to which these articles met the criteria for inclusion in this review. Conflict regarding the eligibility of studies during each stage of the screening process was resolved in discussion with a third reviewer (GI).

*Data Collection:*

A customized data extraction template was created in the Covidence data extraction template. The template included (1) author, (2) year, (3) study design, (4) population characteristics: size and demographic data, (5) type of intervention, (6) comparative groups, and (7) main outcome. Two reviewers (JU and YE) conducted data extraction independently, and a discussion with the third reviewer (GI) was used to obtain a consensus. When there were insufficient details in the manuscript, authors were emailed for further information, especially to check the possibility of meta-analysis.

*Quality assessment of individual studies:*

The quality assessment was performed independently by the two reviewers (JU and YE). The third reviewer (GI) resolved any conflict and disagreements. The Joanna Briggs Institute (JBI) quality assessment criteria, each tailored to a specific study design, were used to critically evaluate the included studies, including “Yes”, “No”, ‘’Unclear”, or “Not Applicable” as options for responding to each parameter in the JBI assessment checklist [22]. The overall risk of bias for each of the included studies was assigned according to the following metrics: a high risk of bias if one or more criteria were assessed as “No”; an unclear risk of bias if one or more criteria were assessed as “Unclear”; and a low risk of bias if all criteria were assessed as “Yes”.

## 3. Results

### 3.1. Study Selection

**Figure 1:**
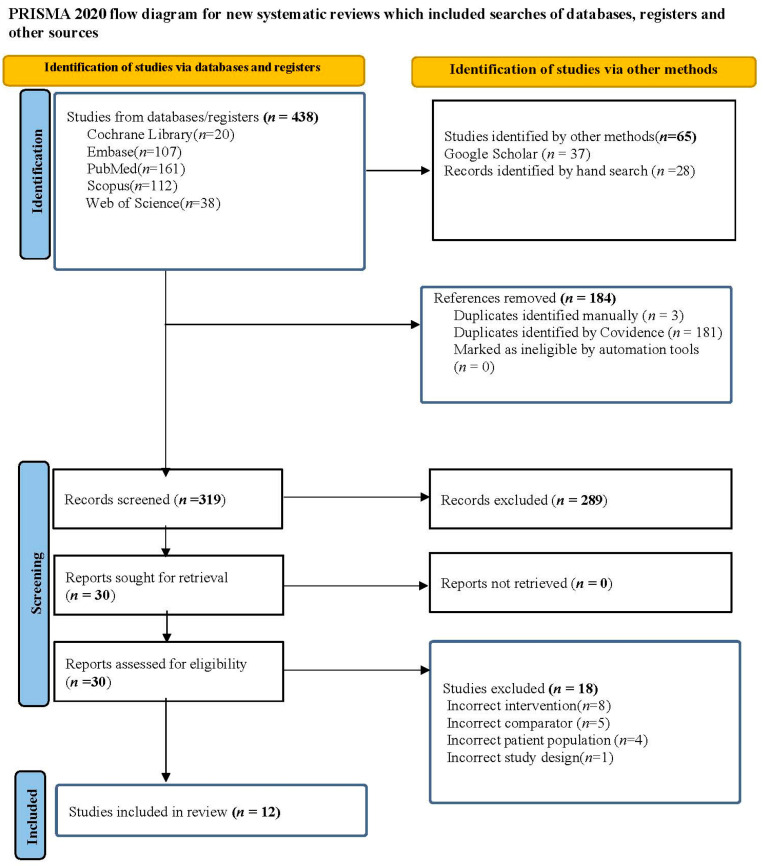
PRISMA flow chart.

The search strategy and selection of the studies are represented in the PRISMA flow diagram (Figure 1). Initially, 475 records were identified from the electronic data search from five databases using the predetermined search criteria. The following is the breakdown of the initial records identified: PubMed, n = 161; Scopus, n = 112; Web of Science, n = 38; Embase, n = 107; Cochrane Library, n = 20; Google scholar, n = 37. Through hand searches, 28 additional records were discovered, bringing the total number to 503 records. In total, 503 references were imported for screening as 503 studies. Overall, 3 duplicates were identified manually, and 181 duplicates were identified via Covidence. A total of 319 studies were screened against the title and abstract, and 289 studies were excluded. Then, 30 studies were assessed for full-text eligibility, and 18 studies were excluded for various reasons (8 for the incorrect intervention being used, 5 for the incorrect comparator being used, 4 for the incorrect patient population being used, and 1 due to the use of the incorrect study design when checked against the inclusion criteria). Twelve studies met the inclusion criteria and were included in this review.

### 3.2. Characteristics of the Included Studies

The characteristics of the included and excluded studies are given in Supplementary Tables S2 and S3. Among the twelve studies selected for this review, four analysed the accuracy of digital impressions in infants with CLP compared to conventional impressions, and all twelve reported the operator’s experience and time related to digital impressions in infants with CLP. By means of a questionnaire, two studies examined the parents’ perspectives on digital impressions. However, a comprehensive overview of all these parameters was not provided individually by any of the studies in the literature.

### 3.3. Risk of Bias in Included Studies

The results of the quality assessment of the included studies are given in Table 1. This review included RCT, cohort studies, cross-sectional studies, case series, and case reports. The JBI critical appraisal tool based on the type of study was used. The quality assessment depended on the number of ‘Yes’, ‘No’, and ‘Unclear’ responses. Among the twelve studies included, three were considered to have a low risk of bias. Five studies had one or more ‘No’ responses in the assessment criteria. As a result, these studies were designated as having a high risk of bias. The remaining four studies had an unclear risk of bias.

### 3.4. Synthesis of Results

#### 3.4.1. Clinician-Centred Outcome

- Accuracy of Digital Impression

The accuracy of digital impressions in infants with CLP was assessed in four studies (Table 2). Among the four studies, two compared the surface discrepancy between digital models from digital impressions and scanned plaster models from alginate impressions. The other two studies evaluated the accuracy of digital impressions by comparing the intra-arch measurements between digital models and scanned plaster models using digital vernier callipers and superimposition.

- Surface Discrepancy

The most substantial surface discrepancy between the models from digital and conventional impressions occurred in the premaxillary segment of infants with bilateral cleft lip and palate (BCLP) [23]. However, no significant difference was found between the two models. This study measured the average deviation of points between the digital images of both impressions, finding a range of 0.42 mm to 0.78 mm. This study also focused explicitly on the premaxillary segment in individuals with BCLP. In this segment, the variations were more pronounced, ranging from +0.46 to −0.46 mm. Conversely, another study evaluated the accuracy of digital impressions in infants by comparing the surface discrepancy between digital and scanned plaster models in an infant with unilateral cleft lip and palate (UCLP) and found no significant difference between both models. The agreement between both models was excellent, with a measured difference of 0.01 mm to 0.1 mm [26].

- Intra-arch measurements

Among the two studies that measured intra-arch measurements, Okazaki et al. observed no significant differences in the dimensions of alveolar cleft defects and alveolar arch width between 3D-printed models from digital impressions and plaster models from conventional impressions [28]. Nevertheless, upon superimposing the digital and scanned plaster models, it was observed that the plaster model group exhibited a greater measured depth of the alveolar cleft defects compared to the intraoral scanner group. A related study by Soliman et al. [29] revealed no statistically significant difference in the intra-arch measurements, such as alveolar cleft defect and alveolar arch width, between 3D-printed models from digital impressions and plaster models from conventional impressions. A meta-analysis was inadequate due to the shortage of data.

- *Operators’ Preference*

Among the twelve studies included in this review, operator experiences during digital impressions in infants were documented in nine studies [9,10,11,12,24,25,26,27,28]. The variables analysed were the difficulties faced by the operators during the digital impression of infants with CLP and the preferred factors such as type of scanners, scanning tips, scanning strategies, and time taken for scanning. Descriptive characteristics of operators’ preferences are given in Table 3. Among those included, one study conducted a comparative analysis of operator experiences with digital and conventional impressions. Dalessandri et al. reported reduced stress associated with digital impressions and less need for retakes [12]. Ten of the included studies reported the time taken for digital impressions in infants with CLP. However, there was no comparison between the duration of digital and conventional impressions in any of the studies.

- *Scanning time*

The average scanning duration in the included studies varied from 80 to 150 s. It has been observed that neonates need more scanning time than infants, and there was no difference in scanning time between awake and anesthetised patients [27].

#### 3.4.2. Patient-Centred Outcome

- *Parents’ perceptions*

Two studies evaluated the parents’ perception regarding digital and conventional impressions in infants with CLP (Table 4). Dalessandri et al. assessed the perception of mothers of infants with CLP undergoing both digital and conventional impression procedures with the help of a questionnaire. Questions were developed and validated to evaluate the psychological effect of the patients’ mothers on the information disclosed about the procedure, their perception of their baby’s suffering during the procedure, and their perception of physical trauma present after taking impressions. After the procedure was completed, further inputs of the mothers’ perceptions of the actual invasiveness of the impression-taking procedure were compared to what they assumed based on the information provided. The response to the questionnaire indicated that mothers of infants with CLP who underwent both digital and conventional impressions preferred digital impressions [12]. Similarly, Soliman et al. showed that the parents of their study had a clear preference for digital impressions [29].

## 4. Discussion

This systematic review critically examined the efficacy of digital impressions in infants with cleft lip and palate (CLP), addressing aspects of accuracy, operator preferences, and parental perceptions.

*Clinician Centred Outcome:*
*Accuracy*
*Key Observations and Challenges*

Two main factors that determine the accuracy of digital impressions are the trueness and precision of measurements. Trueness refers to the closeness of a measured value to the true or reference value. It can be assessed by comparing the measurements obtained from digital impressions to a reference standard of conventional impressions. Precision refers to the consistency or reproducibility of measurements. In the context of digital impressions, repeated measurements may be taken, and the variability between these measurements should be analysed to determine precision [30,31,32]. In the absence of specific information about the methodologies used in the studies, it is challenging to provide precise details about how trueness and precision were measured in the included studies that assessed the accuracy of digital impressions.

Out of the studies included in this review, four specifically assessed the accuracy of digital impressions in infants with cleft lip and palate compared to conventional impressions. These studies employed surface discrepancy or intra-arch measurements or a combination of to evaluate the accuracy of digital impressions. Two of the four studies employed surface discrepancy, one employed intra-arch measurements, and one employed both intra-arch measurements and surface discrepancy. The specific methods and measurements varied among the studies, but collectively, they provided insights into the comparable accuracy of digital and conventional impressions in infants with cleft lip and palate. However, these studies have small sample sizes ranging from one to seven, which might limit the generalisability of their findings. Furthermore, the scientific evidence pertaining to the accuracy noted in this particular context is constrained, as the studies exhibit a high or unclear risk of bias.

Patel et al. observed the most significant variation in surface discrepancy in the premaxillary area of infants with BCLP [23]. This could be attributed to the excessive pressure from impression material in the premaxillary area and subsequent displacement during the conventional impression technique. Moreover, during conventional impression, the accuracy of the impression can be affected by the mobility of the premaxilla, as it is only supported and connected to the vomer bone apically. In addition, obtaining an adequate buccal extension is difficult due to the absence of orbicularis sphincter function; the impression of the premaxillary segment and the cleft area can be challenging due to limited mouth opening and visibility. Meanwhile, digital impressions apply no pressure to oral structures and enable better capturing of the morphology of the oral structure without any displacement. However, digital impressions are not without their limitations.

A comparison of the alveolar cleft depth measurement between digital and conventional impressions in infants revealed a deeper alveolar cleft defect in plaster models compared to digital models [28]. The difficulty of registering the deepest part of the cleft deformity with an intraoral scanner is reported in small children as well [33]. Several factors contribute to this limitation, including the relatively small mouth opening of children, the bulkiness of the scanner head, and the scanner head’s inability to reach the deepest part of the cleft defect. The effect of scanning tip size on achieving accurate digital impressions in infants with CLP is yet to be explored. None of the included studies in this systematic review considered the effect of scanning tips on the accuracy of digital impressions in infants with CLP. Given the limited mouth opening in neonates and infants and the challenge of accessing the deepest part of the cleft deformity with available scanners, it is crucial to investigate how using a small scanning tip affects the accuracy of the digital scan. However, previous studies have shown that small scanning tips can negatively affect both the trueness and precision of digital intraoral scans of the complete dentulous arch when compared to a regular scanning tip [34].

Another factor that affects the accuracy of digital intraoral scans in complete arch digital impressions is the type of scanner used. Among the intraoral scanners used in infants with CLP in this review were the TRIOS Classic (3Shape, Copenhagen, Denmark), the CEREC Omnicom (Sirona Dental Systems, Wals bei Salzburg, Austria), the CS3600 Carestream (Dental, Rochester, NY, USA), and the MEDIT-i500 (Republic of Korea). Most operators preferred the TRIOS Classic (3Shape, Copenhagen, Denmark), due to the scanning speed of 3000 images per second. Amornvit et al., Michelinakis et al., and Kernen compared the accuracy of different scanners, revealing variations in trueness and precision in different scanners in adult dentition [35,36,37]. Notably, among the scanners tested, the Trios series showed the best scan results in terms of accuracy for full arch scanning [36]. However, it is essential to highlight that no comparative studies have analysed the accuracy of different scanners in infants with CLP.

The rehabilitation of cleft lip and palate patients begins shortly after birth, and a multidisciplinary team manages these patients. The orthodontic intervention of cleft lip and palate patients begins with presurgical orthopaedics, such as nasoalveolar moulding, soon after birth [38]. During the preadolescent period, orthodontic treatment mainly aims to coordinate the alveolar segment before bone grafting, which includes interceptive treatment, including expansion, maxillary protraction, and dental alignment. Alongside this is the assessment of surgical timing for possible further grafting and orthognathic surgery. Subsequently, definitive orthodontic treatment with or without orthognathic surgery may take place in the teenage years and adulthood, respectively [38]. Therefore, the process of intraoral scanning of these patients involves scanning the edentulous arch in neonates, partially edentulous arches in infancy, and dentulous arches in the later years. When comparing the accuracy of digital impressions in the complete arch scanning of dentulous and edentulous arches in adults, it has been found that precision is low for dentate scans and particularly low for edentulous scans [39]. Given that the rehabilitation of patients with CLP starts at birth and involves scanning at various stages, including the edentulous stage in neonates, the partially dentulous stage in infancy and the completely dentulous stage in children and adults, it is clear that the accuracy of digital impressions should be studied in each stage. This systematic review failed to identify any such studies and encourages future research in this area.

Different intraoral scanners advocate for various scanning strategies/sequences to ensure proper scanning. In CLP cases, arch discontinuity due to the alveolar cleft poses a unique challenge. When evaluating the effect of different scanning strategies on the accuracy of the complete edentulous scan, it has been found that the scanning strategies impact both the trueness and precision of the digital scans of completely edentulous arches [32,40]. Unfortunately, only two studies included in this review described the scanning strategy used. Special consideration should be given to scanning the alveolar clefts to bridge the gap in patients with CLP before palatal repair, mainly when dealing with deeper and wider clefts. Weise et al. described methods to create a ‘virtual bridge’ between the cleft segments, emphasizing the need for additional critical studies to identify a scanning strategy that can enhance the accuracy of digital impressions in terms of trueness and precision for infants with CLP [25].

*Operator’s experience*

Operators preferred digital impressions over conventional impressions in infants with CLP. The reasons for their preference are multitudinous. Digital impressions register the oral structures to a clinically sufficient level, limit the need for retakes, and increase the ease of registering the missing data without repeating the entire impression procedures. Conventional impressions may lead to a decrease in oxygen saturation levels of around 5% and generally require the presence of an airway management team [6]. The ability to avoid the risk of respiratory obstruction with digital impressions makes it less stressful for the clinicians.

In addition, reducing discomfort for neonates, enabling real-time adjustments, being integrated seamlessly into workflows, and facilitating improved collaboration within multidisciplinary teams could contribute to a more efficient and positive experience for clinicians working with neonates with CLP.

However, operators have faced several difficulties with digital impressions of infants with CLP. A lack of continuity of the arches, small oral cavities, frequent movements of infants, and increased salivation during digital impressions were some of these challenges. In addition, compared to other craniofacial disorders for which digital impressions were used, those with CLP required the most extended scan duration and more repetitions [25].

Innovative techniques have been explored to address the challenges regarding scanning software struggling in cleft regions. In one study, researchers experimented with inserting cotton swabs or using the tip of a glove to bridge the gaps in the cleft segments during scanning, whereas in another study, the handles of bonding brushes were used to bridge the gap [25,26]. By strategically placing these materials, the clinicians aimed to connect the discontinuous parts of the jaw, facilitating more accurate registration in the cases with wider clefts. Additionally, researchers suggested adjusting the scanning pattern to include intact areas of the lip, jaw, palate, or nose as reference points, enhancing the overall accuracy of digital impressions in patients with complex anatomical variations of CLP. The difficulty in registering the deeper part of the cleft was another difficulty faced by the operators. Considering this difficulty and small mouth opening, operators preferred a smaller scanner head [10,11,12,24,26]. There is a clear need for further development of intraoral scanning software algorithms based on different models with CLP to accurately capture the defects both in width and depth.

*Patient-Centred Outcome:*
*Parents’ perceptions*

The quality of life (QoL) of patients is negatively affected by the aesthetic and functional impairments caused by CLP. Parents of children with CLP have a lower quality of life due to increased maternal stress, long-term hospital stays, financial burden, and disruption of social relations [17,41]. Concerning the impact of orthodontic treatment on the oral health-related quality of life (OHRQoL) of parents of cleft children, it has been reported that they have poor OHRQoL compared to parents of non-cleft children [42]. However, these studies did not include questions around digital impressions. In this context, whether the reduced anxiety parents had regarding digital impressions would improve the OHRQoL with CLP-associated orthodontic treatment needs to be explored. Overall, there was a clear preference among parents towards digital impressions, even though this was based on a limited number of studies with small sample sizes and a high risk of bias [12,29].

Patient-reported outcome measures (PROMs) have been extensively studied in different aspects of cleft care. In order to assess PROMs on a global scale, cross-sectionally validated instruments such as the CLEFT-Q scales, Cleft Hearing Appearance and Speech Questionnaire (CHASQ), Child Oral Health Impact Profile (COHIP), and health-related quality of life (HRQL) have been developed [43]. The type of questionnaire used for evaluating parent perceptions regarding digital impressions is an area which would benefit from further refinement. The included studies in this review used a questionnaire adopted from a previous study that evaluated parents’ responses to NAM [44]. The response set to each question was different in two studies. Chlamers et al. used a different type of questionnaire to evaluate the parental perception of adolescents with CLP being submitted to digital impressions [14]. Specific questionnaires based on HRQoL need to be developed and validated for digital impressions and workflow. This will ensure the credibility, reliability, and comparability of data across diverse populations and settings.

The inclusion criteria in our systematic review were designed to focus specifically on studies involving neonates and infants, where conventional impression techniques pose unique challenges. Expanding the criteria for inclusion could have yielded a greater quantity of data for evaluation and review. However, by limiting the inclusion to studies conducted in this population subset, this systematic review aimed to highlight the feasibility and effectiveness of digital impressions at the early stages of cleft lip and palate management, where accurate diagnostic information is crucial for guiding treatment decisions and optimising patient outcomes. By employing this focused methodology, this review aimed to provide valuable insights into the potential utility of digital impressions in improving the quality of care for this vulnerable patient population. While the strict inclusion criteria in the current study limited the number of studies used to draw conclusions, ensuring that our results are transferable to clinical practice is important. Furthermore, relying on broader inclusion criteria would have resulted in including studies that are not comparable due to the inherent differences between methodologies, aims, and objectives. Therefore, the inclusion criteria within our study were informative about the types of studies required to fill the gaps in the current literature and guide future research directions.

Using digital impression techniques in infants with CLP has significant clinical implications. These include patient-centred outcomes that reflect parental perceptions and clinician-centred outcomes that pertain to accuracy and operator preferences. Concerning clinician-centred outcomes, a critical factor to consider is the comparative accuracy of digital and conventional impressions in infants with CLP. The findings of this systematic review, indicating comparable accuracy between digital and conventional impressions in infants with cleft lip and palate (CLP), are consistent with prior research that has indicated the potential of digital impressions in paediatric dentistry [45]. Nevertheless, the precise manner in which scanning techniques, such as the utilisation of a variety of scanners and scanning strategies and the implementation of smaller scanning tips, affect the accuracy of digital impressions remains uncertain, particularly with regard to their effect on the trueness and precision components of accuracy [46,47].

The benefits of digital impressions align with this systematic review’s emphasis on the unanimous preference of both operators and parents for digital impressions. However, the challenges unique to the CLP population, such as disrupted arch continuity and increased movements, increased salivation, and a small oral cavity, emphasise the need for further exploration to optimise digital impression protocols for this specific demographic [10,24,25].

Despite the promising potential of digital impressions, clinicians must interpret the findings cautiously, considering the limitations of the existing evidence base, including the small number of studies and the high risk of bias. Although this review offers significant insights regarding the potential utility of digital impressions in infants with CLP, it also emphasises the necessity for additional research to fill in gaps in knowledge and improve the practicality of these techniques in clinical settings.

Recommendations for clinicians based on the limited findings include

- Adopting digital impression techniques as a feasible option in managing infants with CLP, considering the unanimous preference for digital impressions among both operators and parents.
- Small scanning tips could improve operator comfort and facilitate intraoral scanning in patients with CLP, although further investigation is needed to determine their impact on accuracy.
- Other scanning parameters, such as scanning strategy and types of scanners, which could affect the accuracy need to be explored.
- Participation in future research endeavours, including randomised controlled trials and comparative studies, to explore the accuracy of and parents’ perception toward digital impressions will contribute to the expanding body of evidence and provide guidance for evidence-based practice.

## 5. Limitations

The primary constraints lie in the scarcity of high-quality studies, notably randomised controlled trials, specifically designed to evaluate the accuracy of taking digital impressions of infants with cleft lip and palate.

## 6. Implications for Future Research

In clinical practice, the process of making an impression of patients with cleft lip and palate (CLP), particularly neonates and infants, is laden with challenges. Integrating digital impression and digital models in treatment planning or intervention offers a potential alternative to conventional impression and plaster models. As revealed by this systematic review, the accuracy of digital impressions is comparable to conventional impressions in infants with CLP, a practice that has prevailed for many years. However, clinicians experienced challenges specific to the cleft lip and palate population, including the lack of continuity of arches, frequent head movement, and increased salivation [24,25,26]. The clinician-centred outcomes drawn from this review indicate a preference for small scanning tips, although the impact of this preference on accuracy remains unclear.

Moreover, factors such as scanning strategy, scanner of choice, and scanning tip size may influence the accuracy of intraoral scanning, highlighting the need for further investigation into these variables [35,37,40].

The limited data on parental perceptions also underscore the need for more comprehensive research in this area. The scant evidence on parental perceptions emphasises a crucial gap in understanding the patient-centred outcomes of digital impressions in infants with CLP. To address these challenges and uncertainties, future research should investigate the influence of scanning parameters on accuracy, explore innovative strategies to mitigate the challenges encountered during digital impression in infants with CLP, and conduct high-quality clinical studies such as randomised controlled trials. By addressing these gaps, researchers can enhance the usability and effectiveness of digital impressions in this vulnerable population, ultimately improving the quality of care and treatment outcomes.

## 7. Conclusions

The conclusions of the current review should be interpreted within the limitations of the number and the quality of the included studies.

- Digital impressions are as accurate as conventional impressions in infants with CLP.
- Parents’ perception of digital impressions in infants with CLP is better when compared to conventional impressions.
- Clinicians preferred digital impressions in infants with CLP.
- Future studies should consider better designs to compare the outcome of utilising digital impressions with conventional impressions, including studying a larger cohort of participants.
- Future developmental research may consider developing intraoral scanning technology to match the unique anatomical features and requirements of infants with cleft lip and palate.

## Figures and Tables

**Table 1:** Quality assessment of the included studies.

Author and Year	Study Design	JBI Quality Assessments												Risk of Bias
		1	2	3	4	5	6	7	8	9	10			
Krey, 2018 [9]	Case report	No	Yes	Yes	Yes	Yes	No	Yes	Yes					High Risk
Patel, 2019 [23]	Case report	Yes	Yes	Yes	Yes	Yes	Yes	Yes	Unclear					Unclear
Shanbhag, 2020 [24]	Case report	Yes	Yes	Yes	Yes	Yes	No	Yes	Yes					High Risk
Batra, 2020 [10]	Case series	Yes	Yes	Yes	Yes	Yes	Yes	Yes	Yes	Yes	Yes			Low risk
Gong, 2020 [11]	Case series	No	Yes	Yes	Yes	Yes	Yes	Yes	Yes	No	Unclear			High Risk
Zarean, 2022 [13]	Case series	No	Yes	Unclear	No	No	No	No	No	No	No			High Risk
Weise, 2021 [25]	Case series	Yes	N/A	Yes	Yes	Yes	Yes	Yes	N/A	Yes	N/A			Low risk
ElNaghy, 2022 [26]	Case series	Yes	Yes	Yes	Yes	Yes	Yes	Yes	Unclear	Unclear	Unclear			Unclear
Benitez, 2022 [27]	Cohort study	Yes	N/A	Yes	Yes	Yes	Yes	Yes	Yes	Yes	N/A			Low risk
Okazaki, 2023 [28]	Case control study	Yes	Yes	Yes	Yes	Yes	Unclear	Unclear	Yes	Unclear	Yes			Unclear
Soliman, 2023 [29]	Case control study	Yes	Yes	Yes	Yes	Yes	Unclear	No	No	N/A	Unclear			High Risk
Dalessandri, 2019 [12]	Randomised Clinical trial	Yes	Yes	Yes	Yes	Yes	Unclear	Unclear	Yes	Unclear	Yes	Yes	Yes	Unclear

JBI conducts critical evaluation of studies by providing specialized tools with criteria tailored to different study designs. JBI (https://jbi.global/critical-appraisal-tools, accessed on 21 April 2023). Green: Criteria fully met or minimal risk of bias. Yellow: Criteria partially met or unclear risk of bias. Red: Criteria not met or high risk of bias.

**Table 2:** Accuracy of digital impression (DI) in infants with CLP.

Author Year	Country of the Study	Study Design	Objectives of the Study Is to Assess	Population	Sample Size	Intervention	Scanner	Comparison	Accuracy Measurement	Outcome Measured	Results	Main Outcome
Patel, 2019 [23]	Australia	Case report	Accuracy of DI compared to CI	Male BCLP Infants 12 Weeks	1	Intraoral Scanner	Trios, 3 Shape Dental Systems, Copenhagen, Denmark)	IDM from Alginate impression	Superimposition	Surface discrepancy	The mean range of point deviations is −0.42 mm to +0.78 mm (+2.80 to −2.80 +/− 0.88 STD) with a +0.46 to −0.46 mm (+1.12 to −1.12 +/− 0.48 STD) premaxillary segment.	DI demonstrates comparable accuracy to CI.
ElNaghy, 2022 [26]	USA	Case series	Accuracy of DI compared to CI	Male and female UCLP Infants 4–5 Weeks	2	Intraoral Scanner	Trios, 3 Shape Dental Systems, Copenhagen, Denmark)	IDM from alginate impression	Superimposition	Surface discrepancy	The average deviation of points: 0.01 mm to 0.1 mm	DI demonstrates comparable accuracy to CI.
Okazaki, 2023 [28]	Japan	Case–control study	Accuracy of DI compared to CI	Male and female UCLP Infants 8–24 weeks with a mean age of 15 weeks	7	Intraoral Scanner	Trios, 3 Shape Dental Systems, Copenhagen, Denmark)	IDM from rubber-based impression.	Intra-arch measurements and superimposition	Intra arch measurements: Alveolar cleft defect (a-a’) and Alveolar arch width (b-b’) Superimposition: Alveolar cleft depth	The plaster model group (STL) had deeper alveolar cleft defects measured than the intraoral scanner group (STL), despite no significant differences in intra arch measurements.	DI demonstrates comparable accuracy to CI.
Soliman, 2023 [29]	Egypt	Case–control study	Reliability of DI	Male and female UCLP Infants 1–4 Weeks	7	Intraoral Scanner	Medit i700, Medit Corp., Seoul, Republic of Korea	IDM from alginate impression	Intra-arch measurements and superimposition	Intra arch measurements: Alveolar cleft defect and Alveolar arch width Surface discrepancy	The models showed no significant differences in dimensions between the two groups, but the premaxilla portion showed significant differences under superimposition.	DI demonstrates comparable accuracy to CI.

UCLP: unilateral cleft lip and palate BCLP: bilateral cleft lip and palate. DI: digital impression. CI: conventional impression IDM: Indirect Digital Mo.

**Table 3:** Clinician’s experience on digital impression in infants with CLP.

Study	Study Design	Objectives	Population	Sample Size	Scanner	Parameter Considered	Observations
Krey, 2018 [9]	Case report	Presents digital workflow for the production of palatal plate	BCLP 8–10 weeks old	2	Cerec Omnicam Ortho (Sirona Dental GmbH, Wals bei Salzburg, Austria)	Operator’s experience	Morphology scans can be conducted to a clinically sufficient level, but deep cleft areas cannot be recorded. Preheated scan head is more tolerated than CI.
Patel, 2019 [23]	Case report	To assess arch form using DI in an infant with bilateral CLP	BCLP 12 weeks old Male	1	Trios 3 Shape (3Shape Dental Systems, Copenhagen, Denmark)	Time	The scanning duration was approximately 60 s.
Dalessandri, 2019 [12]	Randomised clinical trial	To evaluate the accuracy, invasiveness, and impact on clinical results of a digital oral impression protocol in the pre-surgical orthopaedic treatment	BCLP and UCLP Infants	3	CS 3600, (Carestream Dental, Atlanta, GA, USA)	Operator’s experience Time	The scanner head, preheated, facilitated scanning, resulting in approximately 30 s of scanning time, with no repetition of DI compared to CI.
Gong, 2020 [11]	Case report	Present full digital workflow to design and manufacturing a consecutive series of customized nasoalveolar molding (NAM)	UCLP Infant	1	Trios 3 Shape (3Shape Dental Systems, Copenhagen, Denmark)	Operator’s experience	The scanning process was facilitated by a small scanner head and faster scanning speed (3000 images per second), but infants showed extreme incoordination during DI.
Shanbhag, 2020 [24]	Case report	Full digital workflow to design and manufacture a consecutive series of customized nasoalveolar molding (NAM)	UCLP 8 weeks	1	Medit 1700 (Medit i700, Medit Corp., Seoul, Republic of Korea)	Operator’s experience Time	The baby’s movement necessitated multiple scans. Preferred Medit scanner to iTero due to its smaller tip, resulting in a 20 min scanning time.
Batra, 2020 [10]	Case series	Presents PSIO treated with a series of clear aligners.	UCLP 1–4 weeks	3	Trios 3 Shape (3Shape Dental Systems, Copenhagen, Denmark)	Operator’s experience Time	“smaller/children” scanning tip was used with a scanning time of 90 to 120 s.
Wiese, 2021 [25]	Case series	To evaluate intraoral scanning (IOS) in infants, neonates, and small children with craniofacial anomalies for its feasibility, scanning duration, and success rate	CLP Trisomy 21 (T21) Robin Sequence (RS) Treacher Collins syndrome (TC) Isolated mandibular retrognathia (MR) Infants and children aged up to 6 years		Trios 3 Shape (3Shape Dental Systems, Copenhagen, Denmark)	Operator’s experience Time	CLP patients face challenges in DI compared to other craniofacial abnormalities, requiring repetition and using objects like cotton swabs or gloves to bridge the cleft gap. Scanning deeper clefts in CLP patients is challenging, with a median duration of 151 s, longer than other conditions. Infants have faster scanning than neonates.
ElNaghy, 2022 [26]	Case series	To evaluate DI technique as a viable alternative to CI in infants with unilateral CLP.	UCLP 4 and 5 weeks old Male and Female	2	Trios 3 Shape (3Shape Dental Systems, Copenhagen, Denmark)	Operator’s experience Time	The scanning process involves using bonding brush handles to bridge the gap, with a small scanner head and faster scanning speed of 3000 images per second. Scanning duration was 80–120 s
Benitez, 2022 [27]	Cohort study	To investigate the implementation and risks of DI for the youngest patients with orofacial clefts	Cleft lip and alveolus UCLP BCLP CPO Neonates Infants Small children Median age 8.7 months	190	Medit 1700 (Medit i700, Medit Corp., Seoul, Republic of Korea)	Operator’s experience Time	The study found no adverse events, no repeat of scans, and no significant difference in scanning time between awake and anaesthesia patients, with cleft type affecting scanning duration. Younger patients need more time for intraoral scanning, with median scanning duration of 85.5 +/− 56 s in cleft palate patients
Zarean, 2022 [13]	Case series	Presents a digital workflow for 3D-printed NAM using intraoral scanner	Not mentioned		Medit 1700 (Medit i700, Medit Corp., Seoul, Republic of Korea)	Time	The time required for scanning varies depending on the complexity of the anatomy, with neonates and infants taking 60 s for cleft palate and 150 s for BCLP.
Okazaki, 2023 [28]	Case–control study	To compare the efficacy of intraoral scanner to that of the conventional plaster model	Male and female UCLP Infants 8–24 weeks with a mean age of 15 weeks	7	Trios 3 Shape (3Shape Dental Systems, Copenhagen, Denmark)	Operator’s experience Time	Infants experienced excessive salivation and shaking during DI, necessitating extensive assistance, and a prolonged period of DI.
Soliman, 2023 [29]	Case–control study	To evaluate the reliability of DI in neonates with cleft lip and palate.	Male and female UCLP Infants 1–4 Weeks	7	Medit 1700 (Medit i700, Medit Corp., Seoul, Republic of Korea)	Time	The scanning time for DI is longer than that of CI, which typically varied from 120 to 150 s.

CLP: cleft lip and palate. UCLP: unilateral cleft lip and palate. BCLP: bilateral cleft lip and palate. CPO: cleft palate only. DI: digital impression. CI: conventional impression. PSIO: presurgical infant orthopaedics.

**Table 4:** Parent’s perception about digital impression in infants with cleft lip and palate.

Author and Year	Country of the Study	Study Design	Objectives of the Study Is to Assess	Population	Sample Size	Intervention	Comparison	Scanner Type	Method of Assessment	Outcome Measured	Observation	Results	Main Outcome
Dalessandri, 2019 [12]	Italy	Case series	Accuracy and invasiveness of DI	BCLP and UCLP Newborns	3	DI	CI	CS3600, Carestream Dental, Atlanta, GA, USA	Questionnaire	Level of concern of mothers after impression procedure explanation Perception of the mother about their child’s suffering during impression procedure Observation of physical trauma after impression taking Perceived invasiveness of the procedure compared to expectation	Level of concern of mothers: 2 (67%) responded as “Not at all” to DI 2 (67%) responded as “Quite a lot” to CI Perception about their child’s suffering during both impression: 2 (67%) responded as “Not at all” to DI 1 (33%) responded as “Slightly” to DI 2 (67%) responded as “Quite a lot” to CI 1 (33%) responded as “A lot” to CI Observation of physical trauma: 2 (67%), responded as “No” with CI 3 (100%) responded as “No” with DI Perceived invasiveness compared to expectation: 1 (33%) responded as “Absolutely less” to DI 2 (67%) responded as “Slightly less” to DI 2 (67%) responded as “Slightly more” to CI 1 (33%) responded as “Much more” to CI	Varying levels of concern and more favourable perception to DI	DI preferred
Soliman, 2023 [29]	Egypt	Case–control study	Reliability of DI	Male and female UCLP Infants with 1–4 Weeks of age	7	DI	CI	Medit i700, Medit Corp., Seoul, Republic of Korea	Questionnaire	Guardian’s acceptance of DI and CI	Level of concern of mothers: 3 (42.9)% responded “yes” to DI 4 (57.1)% responded “yes” to DI 7 (100)% responded “yes” to CI Perception about their child’s suffering during both impressions: 7 (100)% responded “No” to DI 7 (100)% responded “yes” to CI Observationof Physical trauma: 7 (100)% responded “No” to DI 4 (57.1)% responded “yes” to CI 3 (42.9)% responded “No” to CI Perceived invasiveness compared to expectation: 7 (100)% responded “No” to DI 7 (100)% responded “yes” to CI	Varying levels of concern and more favourable perception to DI	DI preferred

UCLP: Unilateral cleft lip and palate. BCLP: bilateral cleft lip and palate. DI: digital impression. CI: conventional impression.

## Data Availability

This article’s data will be shared on request to the corresponding author.

## Supplementary Materials

The following supporting information can be downloaded at: https://www.mdpi.com/article/10.3390/children11030343/s1, Supplementary Table S1: Search Strategy, Supplementary Table S2: Characteristics of included studies. Supplementary Table S3: Characteristics of excluded studies.
