# Supplementary material for: Clinician- and Patient-Centred Outcomes of Digital Impressions in Infants with Cleft Lip and Palate: A Systematic Review
Source: Children (Basel). 2024 Mar 13;11(3):343. doi: 10.3390/children11030343 (PMC10968856; doi:10.3390/children11030343)
Supplement: Supplementary file 1 [file children-11-00343-s001.zip › Supplementary Table S1- search strategy.pdf]

Clinician-and Patient- Centred Outcomes of Digital Impressions in Infants with Cleft Lip and Palate: A Systematic Review

**Database:**	**Search Strategy**
Pubmed	(cleft*[Title/Abstract] AND (lip*[Title/Abstract] OR palate*[Title/Abstract] OR orofacial [Title/Abstract] OR alveolus) [Title/Abstract]) AND ((intraoral AND scan*) OR "3 D Scan*" OR "digital model*"OR "digital impression*""3 D model*"OR "digital workflow*"OR "digital work flow "OR "3D))
Scopus	TITLE-ABS-KEY (("cleft lip and palate" OR "orofacial cleft*" OR "alveolar cleft") AND (" intraoral scan*" OR "3 d scan*" OR "digital impression" OR "digital workflow" OR " 3d print*" OR "computer aided" OR "CAD")) AND (LIMIT-TO (SUBJAREA, "MEDI") OR LIMIT-TO (SUBJAREA, "DENT")) AND (LIMIT TO (LANGUAGE, "English"))
Web of science	ALL (("cleft lip and palate" OR "orofacial cleft*" OR "alveolar cleft") AND ("intraoral scan*" OR "3 d scan*" OR "digital impression" OR "digital workflow" OR " 3d print*" OR "computer aided"))
Cochrane Library	((("cleft lip and palate" OR "orofacial cleft*" OR "alveolar cleft") AND ("intraoral scan*" OR "3 d scan*" OR "digital impression" OR "digital workflow" OR " 3d print*" OR "computer aided" OR "CAD")))
Embase	(cleft*[Title/Abstract] AND (lip*[Title/Abstract] OR palate*[Title/Abstract] OR orofacial [Title/Abstract] OR alveolus) [Title/Abstract]) AND ((intraoral AND scan*) OR "3 D Scan*" OR "digital model*"OR "digital impression*""3 D model*"OR "digital workflow*"OR "digital work flow "OR "3D))
